# Quality of Life and Sense of Coherence in Cancer Patients of German, Turkish and Polish Origin—A Cross-Sectional Study

**DOI:** 10.3390/ijerph20032092

**Published:** 2023-01-23

**Authors:** Marietta Lieb, Yesim Erim, Eva Morawa

**Affiliations:** Department of Psychosomatic Medicine and Psychotherapy, University Hospital of Erlangen, Friedrich-Alexander University Erlangen-Nürnberg (FAU), 91054 Erlangen, Germany

**Keywords:** quality of life, sense of coherence, cancer, migration background, Turkish, Polish, German

## Abstract

Background: Due to a variety of symptoms and side-effects of cancer treatment, quality of life can be considerably impaired in cancer patients. Especially, cancer patients with a migration background seem to be at risk. The objective of our study is to investigate the quality of life and sense of coherence in adult cancer patients of German, Turkish and Polish origin. Methods: We assessed sociodemographic, migration-specific and cancer-related parameters. Quality of life was measured via the EORTC QLQ-C30, sense of coherence was measured with the SOC-13. Results: We included 227 patients in the study (59.5% native Germans, 40.5% with migration background). Native Germans did not differ in quality of life compared to all migrants. However, more nausea and vomiting (NV) and higher appetite loss (AP) was found in Turkish migrants compared to Polish migrants and native Germans. For sense of coherence, we observed significantly higher scores for native Germans compared to all migrants. Turkish migrants had significantly lower scores compared to native Germans. Conclusions: A Turkish migration background seems to play an important role in the perception of symptoms, especially of nausea and vomiting and appetite loss. Culture-specific aspects should be considered in cancer care.

## 1. Introduction

Cancer has a tremendous impact on patients’ lives, including both their physical and psychological well-being. Despite recent advances in cancer treatment, symptoms such as fatigue, nausea, sleep disorders, pain, cognitive dysfunction and many more can affect the daily life of cancer patients, compromising their quality of life (QoL) to a considerable degree [1,2]. In several studies, QoL was found to be markedly impaired in cancer patients when compared to the general population [2,3,4]. Especially, cancer patients with a migration background seem to have a notable drawback when it comes to QoL: in addition to the massive health consequences of cancer, migrants are faced with many difficulties, such as differences in language, culture and religion, discrimination or other post-migration stress factors that can adversely affect QoL [5,6]. A variety of international studies outline an inferior QoL of immigrant cancer patients when compared to native cancer patients [7,8,9].

Political conflicts, unemployment in the home country or other environmental or family reasons have led to an increase of migration in Germany in recent years, which makes it more and more relevant to bear in mind the QoL of migrants, most notably for vulnerable groups such as cancer patients [10]. In 2021, 27.2% of the total population in Germany had a migration background, with Turkish and Polish migrants constituting the two largest migrant groups [11]. Despite the significance of this subject, studies on immigrant cancer patients in Germany seem to be lacking. However, three German studies compared the QoL of healthy migrants of different origin: Morawa and Erim [6] found that Turkish migrants had significantly lower scores in both mental and physical health-related QoL than Polish migrants in Germany, while Buchcik et al. [5] demonstrated that these two migrant groups only differed in some aspects of QoL (general health, vitality, role emotional), but not in others. A third study [12] detected lowest rates of physical QoL for Turkish migrants when compared to migrants of other origin.

A person’s capacity to respond to stressful life events can be depicted with an internationally prominent construct developed by Antonovsky, called sense of coherence (SOC) [13]. There are several studies on adult cancer patients that confirm the relationship between a high SOC and decreased depressive symptoms [14], lower distress [15], more coping strategies [15] and a higher QoL [15,16]. To our knowledge, research on SOC in cancer patients with a migration background is lacking. However, one study from our research group [17] observed that both healthy indigenous and immigrated Poles had lower SOC scores and lower QoL than the German norm, while another study [18] found that both immigrated and local Turkish psychiatric/psychosomatic patients had lower SOC scores than the Turkish and German norm.

So far there is no study in Germany that has investigated the QoL and SOC of cancer patients with different ethnic origin. Based on prior research, we hypothesized a lower QoL and SOC in cancer patients with migration background, especially for those with Turkish origin.

## 2. Materials and Methods

### 2.1. Participants and Data Collection

Cancer patients were recruited via the regional Clinical Cancer Registry Erlangen-Nürnberg which is part of the Bavarian Cancer Registry. Questionnaires were presented in German and Turkish or Polish, respectively, so the migrants could choose the preferred language. Data were collected between April 2016 and October 2017. Inclusion criteria for study participation were as follows: 18 years of age, initial diagnosis of breast-, prostate- or colon cancer or other cancer entities from the clinics of Gynaecology, Urology and Surgery of the University Hospital of Erlangen established between January 2008 and December 2016 as well as country of residence in Germany. Further details on the recruitment process have been reported previously [19]. The study was approved by the ethics committee of the Medical Faculty of the Friedrich-Alexander University Erlangen-Nürnberg (FAU). Project identification code: 232_14B. Written informed consent was obtained from all participants.

### 2.2. Measures

#### 2.2.1. Sociodemographic, Migration-Specific and Cancer-Related Characteristics

For all patients, sociodemographic (gender, age, marital status, partnership, education, employment, household income, religion), migrant-specific (birthplace, length of residence in Germany, age at migration, residence permit, German language proficiency) and cancer-specific parameters (tumor entity, time since diagnosis, metastases, cancer treatment in the last 3 months, psychological/psychiatric treatment) were assessed via self-report questionnaire. In case of missing values regarding clinical parameters, we completed the relevant data from the local tumor center. 

#### 2.2.2. Quality of Life

For the measurement of QoL we used the EORTC QLQ-C30, a validated 30-item questionnaire, specifically designed for the assessment of QoL in cancer patients [20]. In addition, a two-item global health and quality of life scale (QL), it measures QoL-related functions (physical functioning (PF), role functioning (RF), cognitive functioning (CF), emotional functioning (EF), social functioning (SF)), specific symptoms and side-effects (fatigue (FA), pain (PA), appetite loss (AP), insomnia (SL), nausea and vomiting (NV), dyspnoea (DY), constipation (CO), diarrhoea (DI)) and the perceived financial burden resulting from cancer and its treatment (financial difficulties, FI). The items of the global health and QoL scale are scored on a 7-point Likert scale from 1 (very bad) to 7 (excellent) whereas all other items are scored on a 4-point Likert scale from 1 (not at all) to 4 (very much). 

#### 2.2.3. Sense of Coherence

Sense of coherence was measured with the 13-item SOC scale (SOC-13), an internationally applied, reliable and validated instrument, with Cronbach α ranging from 0.70 to 0.92 [21]. All items are rated on a 7-point Likert scale. The total sum score ranges from 13 to 91. 

### 2.3. Data Analysis

Data was processed and analyzed with the software SPSS 28 for Microsoft Windows©. For missing values in the EORTC QLQ-30 and SOC-13, we proceeded as follows: As described in the manual of the EORTC QLQ-30, scales were calculated when at least half of the items from the scale were answered. For all other cases we used expectation maximization (EM) imputations in case of single missing items. If the whole scale was left out, we excluded this person from analysis (*n =* 2). For descriptive statistics, we depicted frequencies, mean values, standard deviations and ranges. We used the Pearson correlation coefficient for computing correlations. To test for differences between the migrant groups and native Germans, we computed ANCOVAs (controlling for age). In case of significant results, we used Bonferroni post-hoc tests to test for group differences. A level of significance of *p* < 0.05 was predetermined for all analyses. 

## 3. Results

### 3.1. Participants

In total, 8200 cancer patients were eligible for participation. Of those, 400 cancer patients were identified as having either a Turkish or Polish migration background (for details please see [19]). We distributed 550 questionnaires to 400 potentially Turkish or Polish and to 150 German cancer patients. Of the 550, 36 patients had either died, had moved or had a cognitive impairment or a comorbid illness. Of the remaining 514 questionnaires, 230 were returned (response rate 45%). We had to exclude additional 3 patients due to exclusion criteria, which resulted in a total of 227 patients. 

### 3.2. Missing Values

For the EORTC QLQ-C30, we observed 0.0% to 1.8% of missing values per item. For the SOC-13, there were 0.9% to 1.8% of missing values. We used EM for imputation, as described in Section 2.3. 

### 3.3. Sample Characteristics

Of the 227 patients, 59.5% (*n =* 135) were native Germans and 40.5% (*n =* 92) had a migration background: 13.2% (*n =* 30) had a Turkish and 11.9% (*n =* 27) a Polish migration background, while 15.4% (*n =* 35) had a migration background classified as ‘other’. Our sample predominantly consisted of female cancer patients (72.2%, *n =* 164). The most common diagnosis was breast cancer (34.3%, *n =* 78). Further sociodemographic, migration and cancer specific data can be found in Table 1. 

Pre-analyses showed a significant difference in age between native Germans and the total migrant group (*p* = 0.012). An ANOVA that compared native Germans, Polish and Turkish patients also revealed a significant age effect (*p* = 0.01) while post-hoc tests detected a difference in age between native Germans and Turkish patients (*p* = 0.025). The respective groups did not differ concerning gender (Germans vs. Migrants: *p* = 0.888; Germans vs. Turkish vs. Polish: *p* = 0.698). We therefore only included age as a control variable in all subsequent analyses. 

### 3.4. Quality of Life

#### 3.4.1. Comparison between Native Germans and All Migrants

When we compared the subscales of the EORTC QLQ-C30 between native Germans and all migrants in total, we found no significant differences between the two groups: QL: *p* = 0.259, η^2^ = 0.006; PF: *p* = 0.256, η^2^ = 0.006; RF: *p* = 0.651, η^2^ = 0.001; EF: *p* = 0.317, η^2^ = 0.004; CF: *p* = 0.234, η^2^ = 0.006; SF: *p* = 0.810, η^2^ < 0.001; FA: *p* = 0.093, η^2^ = 0.013; NV: *p* = 0.322, η^2^ = 0.004; PA: *p* = 0.801, η^2^ < 0.001; DY: *p* = 0.233, η^2^ = 0.006; SL: *p* = 0.900, η^2^ < 0.001; AP: *p* = 0.196, η^2^ = 0.007; CO: *p* = 0.226, η^2^ = 0.007; DI: *p* = 0.587, η^2^ = 0.001; FI: *p* = 0.201, η^2^ = 0.007. 

#### 3.4.2. Comparison between Native Germans, Turkish and Polish Migrants

In order to examine specific differences in QoL between native Germans, Turkish and Polish patients, we excluded the migrants labeled as ‘Other’. For Global Health Status (QL) and all functioning scales, there were no significant differences between German, Turkish or Polish patients: QL: *p* = 0.884, η^2^ = 0.001; PF: *p* = 0.143, η^2^ = 0.020; RF: *p* = 0.639, η^2^ = 0.005; EF: *p* = 0.189, η^2^ = 0.017; CF: *p* = 0.263, η^2^ = 0.014; SF: *p* = 0.897, η^2^ = 0.001. 

For the symptom scales, however, we found a significant main effect of migration status for nausea and vomiting (NV): F(2188)= 5.684, *p* = 0.004, η^2^ = 0.057. Post-hoc tests revealed significantly more NV in Turkish migrants when compared to German natives (13.53, 95%CI [2.99, 24.06], *p* = 0.007) as well as Polish migrants (16.63, 95%CI [3.04, 30.23], *p* = 0.011). There was no difference between native Germans and Polish migrants (3.11, 95%CI [−0.7.80, 14.02], *p* = 1.00). We further found a significant effect of migration status on appetite loss (AP): (F(2188) = 5.556, *p* = 0.005, η^2^ = 0.056). Post-hoc tests showed that Turkish migrants had a significantly higher AP than Germans (15.28, 95%CI [1.40, 29.16], *p* = 0.025) and Polish migrants (23.91, 95%CI [5.99, 41.82], *p* = 0.004). German and Polish patients, however, did not differ significantly (8.6, 95%CI [−5.76, 23.00], *p* = 0.447). 

Although many symptom scores were higher in Turkish migrants in relation to German and Polish patients, no significant effects were revealed in the analyses for all other symptoms: FA: *p* = 0.139, η^2^ = 0.021; PA: *p* = 0.995, η^2^ < 0.001; DY: *p* = 0.250, η^2^ = 0.015; SL: *p* = 0.546, η^2^ = 0.006; CO: *p* = 0.125, η^2^ = 0.022; DI: *p* = 0.653, η^2^ = 0.005; FI: *p* = 0.653, η^2^ = 0.005. The data of the EORTC QLQ-C30 for each group are presented in detail in Table 2.

### 3.5. Sense of Coherence

#### 3.5.1. Comparison between Native Germans and All Migrants

We found a significant difference between the sum score of SOC-13 of native Germans (M = 64.92, SD = 12.18) and the total group of migrants (M = 59.14, SD = 11.87), with native Germans scoring significantly higher: F(1222) = 9.23, *p* = 0.003, η^2^ = 0.040. 

#### 3.5.2. Comparison between Native Germans, Turkish and Polish Migrants

**Figure 1:**
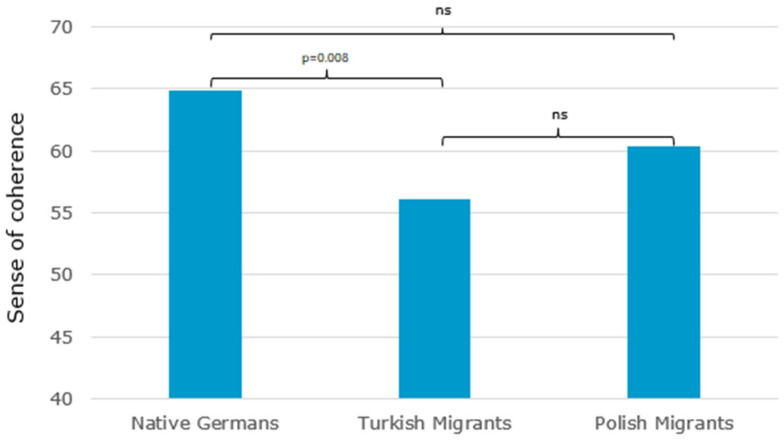
Mean values for sense of coherence: comparison between cancer patients of German, Turkish and Polish origin.

When we examined native Germans (M = 64.92, SD = 12.18), Turkish (M = 56.07, SD = 13.37) and Polish migrants (M = 60.33, SD = 9.13) as separate groups, we found a significant main effect: F(2186) = 4.99, *p* = 0.008, η^2^ = 0.051. Post-hoc tests revealed significantly lower SOC in patients with Turkish origin when compared to native Germans (−7.39, 95%CI [−13.23, −1.56], *p* = 0.008). There were no significant differences between patients of Turkish and Polish origin (−3.94, 95%CI [−11.46, 3.58], *p* = 0.622), nor between Polish patients and native Germans (−3.45, 95%CI [−9.50, 2.59], *p* = 0.508). Please see Figure 1. 

### 3.6. Correlation between Sense of Coherence and Quality of Life in the Different Migrant Groups

We correlated the sum score of SOC-13 with the global health and quality of life scale (QL) of the EORTC QLQ-C30 for the different groups. The results are displayed in Table 3.

## 4. Discussion

To our knowledge, this is the first study that investigated the QoL and SOC in cancer patients of different origin in Germany, with a specific focus on patients with Turkish and Polish migration background. 

In contrast to international studies, we did not find any difference in QoL, when we compared native Germans with the total migrant group [7,8,9]. However, in the review of Luckett et al. [7], inferior QoL was only found for Hispanic cancer patients when compared to native U.S. patients, while this difference could not consistently be found for other ethnic groups in specific domains of QoL (e.g., emotional, social, functional). Migrants are an extremely heterogeneous group regarding language, culture and religion, which can lead to different risk and protective factors [12] and, thus, to potential disparities in QoL. Differences within the migrant group could cancel each other out, which is why it is crucial to differentiate between different ethnicities: When we performed more detailed analyses that compared native Germans and patients with Turkish and Polish origin we found that Turkish migrants suffer significantly more from nausea and vomiting as well as higher appetite loss. Although most of the other symptoms were also highest and most of the functional scales lowest for Turkish patients, the differences between the three ethnic groups were not statistically significant, possibly due to limited power. Still, it seems that different ethnicities do not differ in all categories for QoL equally, but only in some: Buchcik et al. [22] found that migrants had significantly lower mental health related QoL than German natives, whereas no difference was found for physical health related QoL. Another study revealed that Turkish migrants only showed lower scores in emotional QoL and mental health when compared to native Germans [5]. However, different instruments were used for measuring QoL. Lim et al. [9] applied the EORTC QLQ-C30 to Asians and Caucasians in Australia. Comparable to our results, Asian migrants also suffered more from nausea and vomiting than natives, whereas no significant difference was found for appetite loss. 

Our results might be explained by a culturally different symptom perception and disclosure of complaints: cancer symptoms are highly subjective phenomena and their perception can vary greatly in different cultures [23,24]. Erbil et al. [24] suggest that especially in Mediterranean countries, somatic complaints are a well-known way to express psychological distress. Research confirms higher somatization in both Turkish cancer patients and healthy migrants of Turkish origin than other ethnicities [24,25]. Especially for a culture where food plays an essential role for culture and social interactions [26], food-related symptoms might be particularly pronounced, such as loss of appetite and nausea and vomiting. Differing cultures also come along with diverging expectations. According to Nestoriuc et al. [27], negative expectations can also increase the risk for side-effects and certain symptoms, which might be the case for Turkish migrants. However, further research is necessary in this field. 

For SOC, we observed significantly higher scores in native Germans when compared to the total migrant group. When comparing native Germans, Turkish and Polish patients, we found that Turkish patients had a significantly lower SOC than native Germans. This coincides with previous results that demonstrated lower SOC scores for immigrated Turkish psychosomatic patients when compared to native Germans [18]. However, norm values for the Turkish population seem to be generally lower than those of Germans [18]. The authors explain this phenomenon with a culture-specific effect: The Turkish culture has a more collectivistic orientation, thus, Turkish patients may attribute skills measured via SOC more to their families than to themselves. This explanation might also apply for the next result: Comparable to previous research, our results confirm the association between higher SOC and better QoL in cancer patients [15,16]. However, the association was highest in patients with Turkish origin (r = 0.728). Especially in Turkish patients, higher SOC scores seem to play an important role for high QoL, whereas in other ethnic groups this association is attenuated. SOC seems to associate with QoL in a more enhanced way in collectivistic cultures than it does in individualistic ones. However, further research is necessary to investigate this phenomenon. 

### 4.1. Study Strenghts and Limitations

The primary strength of our study is that it includes the two most numerous ethnic groups in Germany (Turkish and Polish migrants). Moreover, migrants with insufficient language proficiency could be recruited due to the use of bilingual questionnaires. A noteworthy limitation of our study is the small sample size of patients of Polish and Turkish origin. Due to limited statistical power, small effects might not have been detected. Further, the small sample size as well as the monocentric design of our study and the unequal distribution of gender might compromise the transferral of our results to other parts of Germany. We further noted a difference in age and time since diagnosis in our sample between native Germans and the migrant groups. Younger migrants with a shorter time since diagnosis could be more willing to participate in studies, which might bias our results and also limit generalizibility. Future studies should involve larger and more representative samples (e.g., age and gender-matched samples) of migrants when comparing them to the native population.

### 4.2. Conclusions

Cancer patients of different origin differ in at least some aspects of QoL. Especially, a Turkish migration background seems to play a role in the symptom perception in cancer, mainly nausea and vomiting and appetite loss. Moreover, resources such as SOC are lowest in this group. In cancer care, differing symptoms and resources should be considered when treating patients with migration background, especially a Turkish one. Culture-specific aspects should be considered in cancer care. However, further research is necessary for more specific implications.

## Figures and Tables

**Table 1:** Sociodemographic, migration and cancer specific data.

Variable	German Natives (*n =* 135)	Turkish Migrants (*n =* 30)	Polish Migrants (*n =* 27)	All Migrants (Turkish + Polish + Other) (*n =* 92)
Age				
M (SD)	59.64 (14.26)	51.97 (14.82)	53.70 (14.11)	55.32 (13.87)
Range	18–87	26–74	19–77	19–80
Gender *n* (%)				
Women	98 (72.6)	23 (76.6)	18 (66.7)	66 (71.7)
Men	37 (27.4)	7 (23.3)	9 (33.3)	26 (28.3)
Marital Status *n* (%)				
Single	13 (9.6)	2 (6.7)	5 (18.5)	11 (12.0)
Married	100 (74.1)	25 (83.3)	16 (59.3)	65 (70.7)
Separated/Divorced	17 (12.6)	2 (6.7)	4 (14.8)	12 (13.0)
Widowed	5 (3.7)	1 (3.3)	2 (7.4)	4 (4.3)
Partnership *n* (%)				
Yes	99 (73.3)	26 (86.7)	19 (70.4)	74 (80.4)
No	32 (23.7)	4 (13.3)	8 (29.6)	18 (19.6)
No data	4 (3.0)	--	--	--
Education *n* (%)				
Below secondary school	100 (74.1)	20 (66.7)	7 (25.9)	45 (48.9)
Above secondary school	34 (25.2)	10 (33.3)	20 (74.1)	47 (51.1)
No data	1 (0.7)	--	--	--
Employment Status *n* (%)				
Employed (full/part-time)	47 (34.8)	12 (40.0)	12 (44.4)	40 (29.3)
Pensioner	70 (51.8)	10 (33.3)	10 (37.0)	33 (35.9)
Student	3 (2.2)	--	2 (7.4)	2 (2.2)
Household	9 (6.7)	4 (13.3)	1 (3.7)	6 (6.5)
Jobseeking	2 (1.5)	3 (10.0)	2 (7.4)	8 (8.7)
Other	4 (3.0)	--	--	2 (2.2)
No data	--	1 (3.3)	--	1 (1.1)
Monthly household income *n* (%)				
<500€	3 (2.2)	2 (6.7)	2 (7.4)	6 (6.5)
500–1000€	16 (11.9)	9 (30.0)	9 (33.3)	23 (25.0)
1000–2000€	35 (26.9)	9 (30.0)	8 (29.6)	29 (31.5)
2000–3000€	40 (29.6)	7 (23.3)	3 (11.1)	20 (21.5)
>3000€	36 (26.7)	3 (10.0)	5 (18.5)	11 (12.0)
No data	5 (3.7)	--	--	3 (3.3)
Religion *n* (%)				
Roman Catholic	52 (38.5)	--	24 (88.9)	36 (39.1)
Protestant	52 (38.5)	--	1 (3.7)	9 (9.8)
Muslim	--	26 (86.7)	--	31 (33.7)
Other	3 (2.2)	1 (3.3)	--	2 (2.2)
None	28 (20.7)	3 (10.0)	2 (7.4)	14 (15.2)
Born in Germany *n* (%)				
Yes	133 (98.5)	5 (16.7)	2 (7.4)	21 (22.8)
No	2 (1.5) *	25 (83.3)	25 (92.6)	71 (77.2)
Length of Residence in Germany (years)				
M (SD)	--	34.9 (11.96)	25.2 (9.24)	29.79 (12.11)
Range	--	5–48	2–36	2–52
Age at migration (years)				
M (SD)	--	20.20 (8.71)	30.77 (9.64)	27.52 (11.45)
Range	--	0–38	3–50	0–58
Residence Permit Status *n* (%)				
German citizenship	128 (94.8)	8 (26.7)	21 (77.8)	56 (60.9)
Other citizenship	--	2 (6.7)	--	3 (3.3)
Dual citizenship	--	--	--	--
Unlimited residence permit status	--	19 (63.3)	5 (18.5)	29 (31.5)
Other	--	1 (3.3)	1 (3.7)	3 (3.3)
No data	7 (5.2)	--	--	1 (1.1)
Language Proficiency *n* (%)				
German as mother tongue	135 (100)	--	1 (3.7)	19 (20.7)
Very good	--	9 (30.0)	7 (25.9)	20 (21.7)
Good	--	2 (6.7)	8 (29.6)	18 (19.6)
Moderate	--	13 (43.3)	7 (25.9)	24 (26.1)
Little	--	5 (16.7)	3 (11.1)	8 (8.7)
None	--	1 (3.3)	--	2 (2.2)
No data	--	--	1 (3.7)	1 (1.1)
Tumor Entity *n* (%)				
Lip, oral cavity, pharynx (C00–C14)	2 (1.5)	--	--	1 (1.1)
Digestive organs (C15–C26)	21 (15.6)	4 (13.3)	5 (18.5)	13 (14.1)
Respiratory system (C30–C39)	3 (2.2)	--	--	1 (1.1)
Bone and articular cartilage (C40–C41)	1 (0.7)	--	--	1 (1.1)
Skin (C43–C44)	8 (5.9)	--	3 (11.1)	6 (6.5)
Soft tissue (C45–C49)	4 (3.0)	--	4 (14.8)	5 (5.4)
Breast (C50)	47 (34.8)	12 (40.0)	5 (18.5)	31 (33.7)
Female genital organs (C51–C58)	23 (17.0)	4 (13.3)	3 (11.1)	10 (10.9)
Male genital organs (C60–C63)	17 (12.6)	4 (13.3)	2 (7.4)	11 (12.0)
Urinary tract (C64–C68)	3 (2.2)	1 (3.3)	1 (3.7)	2 (2.2)
Thyroid and endocrine gland (C73–C75)	2 (1.5)	2 (6.7)	--	3 (3.3)
Hematologic (C80–C96)	4 (3.0)	3 (10.0)	4 (14.8)	8 (8.7)
Time since diagnosis (months)				
M (SD)	45 (23.82)	33.67 (22.84)	35.37 (22.99)	35.85 (22.63)
Range	6–90	3–102	7–75	3–102
Metastases *n* (%)				
Yes	32 (23.7)	4 (13.3)	4 (14.8)	13 (14.1)
No	103 (76.3)	26 (86.7)	23 (85.2)	79 (85.9)
Cancer treatment in the last 3 months *n* (%)				
Yes	61 (46.6%)	16 (53.3%)	13 (50.0%)	44 (49.45)
No	70 (53.4%)	14 (46.7%)	13 (50.0%)	45 (50.6%)
Prior psychological/psychiatric treatment *n* (%)				
Yes	27 (20.0)	8 (26.7)	7 (25.9)	20 (21.7)
No	102 (75.6)	22 (73.3)	19 (70.4)	67 (72.8)
No data	6 (4.4)	--	1 (3.7)	5 (5.4)
Current psychological/psychiatric treatment *n* (%)				
Yes	10 (7.4)	5 (16.7)	1 (3.7)	11 (12.0)
No	113 (83.7)	23 (76.7)	23 (85.2)	73 (79.3)
No data	12 (8.9)	2 (6.7)	3 (11.1)	8 (8.7)

Note: * Native German resettlers from former German territories (=”Vertriebene”).

**Table 2:** Results of the EORTC QLQ-C30.

		Native Germans (*n =* 135)	Turkish Migrants (*n =* 30)	Polish Migrants (*n =* 27)	All Migrants (Turkish + Polish + Other) (*n =* 92)
Global Health Status/Quality of Life (QL)		63.99 (23.18) 0–100	63.79 (27.03) 0–100	62.96 (17.80) 25–100	61.36 (23.19) 0–100
Functional Scales	Physical Functioning (PF)	76.83 (23.55) 0–100	71.33 (18.87) 40–100	80.00 (17.25) 46.67–100	75.02 (20.60) 20–100
	Role Functioning (RF)	64.32 (32.27) 0–100	67.22 (31.71) 0–100	71.60 (31.63) 0–100	67.03 (31.35) 0–100
	Emotional Functioning (EF)	60.39 (26.36) 0–100	49.91 (34.99) 0–100	62.35 (22.81) 16.67–100	55.71 (28.96) 0–100
	Cognitive Functioning (CF)	76.99 (22.92) 0–100	67.22 (35.42) 0–100	72.22 (26.55) 16.67–100	71.92 (29.12) 0–100
	Social Functioning (SF)	61.82 (32.63) 0–100	63.33 (36.20) 0–100	64.20 (36.89) 0–100	62.68 (34.18) 0–100
Symptom Scales	Fatigue (FA)	41.48 (28.11) 0–100	53.70 (33.66) 0–100	44.86 (28.49) 0–100	48.55 (31.37) 0–100
	Nausea and Vomiting (NV)	9.38 (19.27) ^a^ 0–100	22.78 (31.71) ^ab^ 0–100	6.17 (14.73) ^b^ 0–66.67	12.32 (23.03) 0–100
	Pain (PA)	37.41 (32.37) 0–100	36.11 (33.36) 0–100	37.04 (31.12) 0–100	37.68 (32.58) 0–100
	Dyspnoea (DY)	29.10 (33.56) 0–100	26.67 (32.04) 0–100	16.05 (28.30) 0–100	22.46 (30.10) 0–100
	Insomnia (SL)	44.69 (35.54) 0–100	48.88 (39.86) 0–100	39.51 (37.02) 0–100	43.84 (38.26) 0–100
	Appetite Loss (AP)	15.06 (26.93) ^a^ 0–100	30.00 (39.49) ^ab^ 0–100	6.17 (13.19) ^b^ 0–33.33	19.93 (30.88) 0–100
	Constipation (CO)	14.43 (26.32) 0–100	24.44 (31.48) 0–100	14.81 (26.69) 0–100	18.47 (28.97) 0–100
	Diarrhea (DI)	15.80 (26.65) 0–100	16.67 (22.74) 0–66.67	11.11 (20.67) 0–66.67	13.92 (22.26) 0–66.67
	Financial Difficulties (FI)	24.88 (31.58) 0–100	33.33 (41.98) 0–100	24.69 (30.09) 0–100	32.25 (36.47) 0–100

Note: Table displays mean (standard deviation) and range. ^a,b^ Groups with the same marks significantly differ on a significance level of *p* < 0.05.

**Table 3:** Correlations between sense of coherence and quality of life.

	Global Health Status/Quality of Life (QL)				
	Total Sample (*n =* 227)	Native Germans (*n =* 135)	Turkish Migrants (*n =* 30)	Polish Migrants (*n =* 27)	All Migrants (Turkish + Polish + Other) (*n =* 92)
Sense of coherence (SOC-13)	r = 0.344 **	r = 0.221 *	r = 0.728 **	r = 0.436 *	r = 0.514 **

Note: * *p* < 0.05, ** *p* < 0.01.

## Data Availability

The data supporting our findings can be requested from PD Dr. rer. medic. Dr. habil. med. Eva Morawa (Eva.Morawa@uk-erlangen.de).
